# Double-Positive CD21+CD27+ B Cells Are Highly Proliferating Memory Cells and Their Distribution Differs in Mucosal and Peripheral Tissues

**DOI:** 10.1371/journal.pone.0016524

**Published:** 2011-01-27

**Authors:** Arpita Das, Huanbin Xu, Xiaolei Wang, Canddy L. Yau, Ronald S. Veazey, Bapi Pahar

**Affiliations:** 1 Division of Microbiology, Tulane National Primate Research Center, Tulane University School of Medicine, Covington, Louisiana, United States of America; 2 Division of Comparative Pathology, Tulane National Primate Research Center, Tulane University School of Medicine, Covington, Louisiana, United States of America; 3 Department of Biostatistics, School of Public Health and Tropical Medicine, Tulane University, New Orleans, Louisiana, United States of America; The University of Hong Kong, Hong Kong

## Abstract

**Background:**

Several B-cell defects arise in HIV infected patients, particularly in patients with chronic infection and high viral load. Loss of memory B cells (CD27^+^ B cells) in peripheral blood and lymphoid tissues is one of the major B cell dysfunctions in HIV and simian immunodeficiency virus (SIV) infection. Despite several studies, definitive identification of memory B cells based on CD27 surface expression has not been described. Similarly, the rates of cell turnover in different B cell subpopulation from lymphoid and mucosal tissues have not been well documented. In this study, we demonstrate the presence of memory B cell populations and define their distribution, frequency and immunophenotype with regards to activation, proliferation, maturation, and antibody production in normal rhesus macaques from different lymphoid tissues.

**Methodology/Principal Findings:**

Thirteen healthy, uninfected rhesus macaques were selected for this study. CD20^+^ B cells were isolated from peripheral blood and sorted based on CD27 and CD21 surface markers to define memory B cell population. All the B cell subpopulation was further characterized phenotypically and their cell turnover rates were evaluated *in vivo* following bromodeoxyuridine (BrdU) inoculation. Double positive (DP) CD21^+^CD27^+^ B cells in both peripheral and lymphoid tissues are memory B cells, able to produce antibody by polyclonal activation, and without T cell help. Peripheral and lymphoid DP CD21^+^CD27^+^ B cells were also able to become activated and proliferate at higher rates than other B cell subpopulations. Increased turnover of tonsillar memory B cells were identified compared to other tissues examined.

**Conclusions/Significance:**

We suggest that this DP memory B cells play a major role in the immune system and their function and proliferation might have an important role in HIV/SIV mediated B cell dysregulation and pathogenesis.

## Introduction

Immunological memory is a crucial feature of adaptive immunity, whereby the first encounter with a pathogen is imprinted indelibly into the immune system [1]. Memory B cells and long-lived plasma cells are responsible for the long-term humoral immunity elicited by most vaccines [2], [3]. Immune responses to T cell-dependent antigen occur within secondary lymphoid tissues. After exposure to a T cell-dependent antigen, naïve B cells can differentiate either rapidly differentiating short-lived immunoglobulin secreting cells or long-lived plasma cells or memory B cells [4], [5], [6], [7]. These newly generated memory B cells can re-enter the circulation or remain as resident cells within discrete regions of secondary lymphoid tissue, like marginal zone of spleen or mucosal epithelium of tonsil [5], [8], [9], [10], [11]. Most of the memory B cells information has come from human studies. Presumably because of the constant exposure to antigens, humans have an abundance of memory-like cells, as defined by the marker CD27 [9], [12], [13]. Surface receptor CD27, a type 1 glycoprotein and a member of tumor necrosis factor receptor family was first reported on a subset of human B cells and was thought that their expression may be acquired late during B cell differentiation [13], [14]. Upon in vitro stimulation by *Staphylococcus aureus* Cowan strain plus interleukin 2, CD27^+^ B cells, in contrast to CD27- B cells, are quickly activated and can produce higher levels of immunoglobulins like IgA, IgM and IgG [12], [15]. In contrast, naïve (CD27-) B cells usually require three different signals to be activated: signal delivered by antigen through B cell receptor; signal delivered by antigen specific T-helper cells via CD40, and signal delivered by microbial products acting on toll-like receptors [16]. Moreover, memory B cells can be activated to proliferate and differentiate into antibody secreting cells in an antigen-independent fashion by microbial products, cytokines, bystander T-cell help, and, possibly other stimuli yet to be defined [3], [16]. Complement receptor 2 (CD21) is a cell-surface protein that contains a small cytoplasmic domain and an extracellular domain consisting of a series of short consensus repeats termed complement control protein domains. CD21, which recognize activated products of complement 3 is predominantly expressed on mature B cells and follicular dendritic cells [17] and is an important receptor for uptake and retention of immune complexes. In the absence of CD21 expression, survival of memory B cells is markedly impaired [18]. HIV-induced immune dysfunction includes B-cell activation and the impaired production of antibodies that is partially related to memory B cells [19], [20], [21], [22], [23]. The cell surface CD27 molecule has been utilized as the major B cell memory marker to examine events in HIV/SIV infection [20], [23], [24], [25]. Similarly, the presence of low level of peripheral CD21^+^ B cells in HIV viremic patients also suggest that impaired function of CD21^+^ B cells may be responsible for HIV mediated immune-dysfunction [20], [21], [26]. Therefore, CD27 and CD21 positive B cells might have an important role in regulating HIV/SIV pathogenesis and generating potent memory B cell responses.

Despite all these studies, identification of specific memory B cell subsets based on CD27 and CD21 surface expression and antibody production has not been well described. Similarly, the rate of cell turnover of B cell subpopulations in different lymphoid and mucosal tissues has not been examined in vivo. In this study, we performed an extensive analysis to characterize memory B cells obtained from peripheral blood (PB), axillary lymph node (ALN), bronchoalveolar lavage (BAL), bone marrow (BM), spleen, tonsil and intestinal lamina propria lymphocytes (LPL) of the jejunum from normal rhesus macaques (RMs). We have characterized memory B cell populations including their distribution, frequency, and immunophenotype with regards to activation, proliferation and maturation in normal macaques. Furthermore, we examined the levels of turnover of different B cell subsets in lymphoid tissues to determine tissue specific B cell proliferation in RMs.

This study shows that double positive (DP) CD21^+^CD27^+^ B cells are memory B cells capable of antibody production by polyclonal activation, and without the help of T cells. Memory CD21^+^CD27^+^ B cells were predominant in all lymphoid tissues except for PB and BM. DP CD21^+^CD27^+^ B cells were also able to activate and proliferate at higher rates than other B cell subpopulations. In addition, these DP cells may be important in HIV infection and pathogenesis due to their high cell turnover rate and increased antibody production.

## Results

### CD21 expression differs between peripheral blood and lymphoid tissue B cells

**Figure 1:**
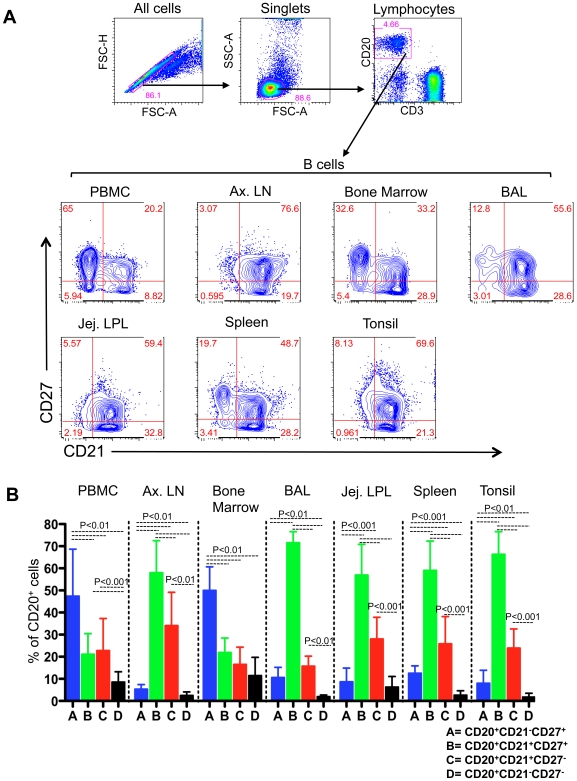
Phenotyping B cell subsets in tissues of rhesus macaques. (**A**) A representative dot and contour plots showing distribution of CD27 and CD21 phenotypes on CD20^+^ B cells from a normal uninfected healthy rhesus macaque (BB01). Singlets were gated first to eliminate doublets and finally gating was performed on CD20^+^ B lymphocytes. All CD20^+^ B cells were further analyzed based on their CD21 and CD27 surface molecule expression. Each quadrant shows percentages of specified populations. (**B**) Mean frequencies (± standard deviation) of single positive (SP) CD27^+^, SP CD21^+^, double positive (DP) CD21^+^CD27^+^ and double negative CD21^−^CD27^−^ B cell subsets are shown as bars for different tissues from uninfected rhesus macaques. Note that in lymphoid tissues including lymph node, bronchoalveolar lavage, spleen, tonsil and jejunum LPL there were increased percentages of DP CD21^+^CD27^+^ B cells compared to peripheral blood and BM B lymphocytes. Statistical significant differences between each group of cells are shown.

In order to determine the distribution of CD21 surface marker in relation to CD27 expression, we performed extensive analysis of B cells from PB and different lymphoid tissues in normal RMs. The frequencies of single positive (SP) CD27^+^, SP CD21^+^, DP CD21^+^CD27^+^ and double negative CD27^−^CD21^−^ B cells were determined in PB, ALN, BM, BAL, spleen, tonsil and jejunum LPL of 13 normal RMs by flow cytometry (Fig. 1A & B). The ratio of SP CD27^+^ to DP CD21^+^CD27^+^ B cells was 2.2∶1 in PB, 0.1∶1 in ALN, 2.3∶1 in BM, 0.1∶1 in BAL, 0.1∶1 in jejunum LPL, 0.2∶1 in spleen and 0.1∶1 in tonsil. Significantly higher percentages of DP CD21^+^CD27^+^ B cells were detected in LN, BAL, jejunum LPL, spleen and tonsil (mean value range from 56.9 to 71.6%) compared to PB (mean value 21.2%) and BM (mean 22%; p<0.05).

### Double positive CD21^+^CD27^+^ B cells are memory B cells

**Figure 2:**
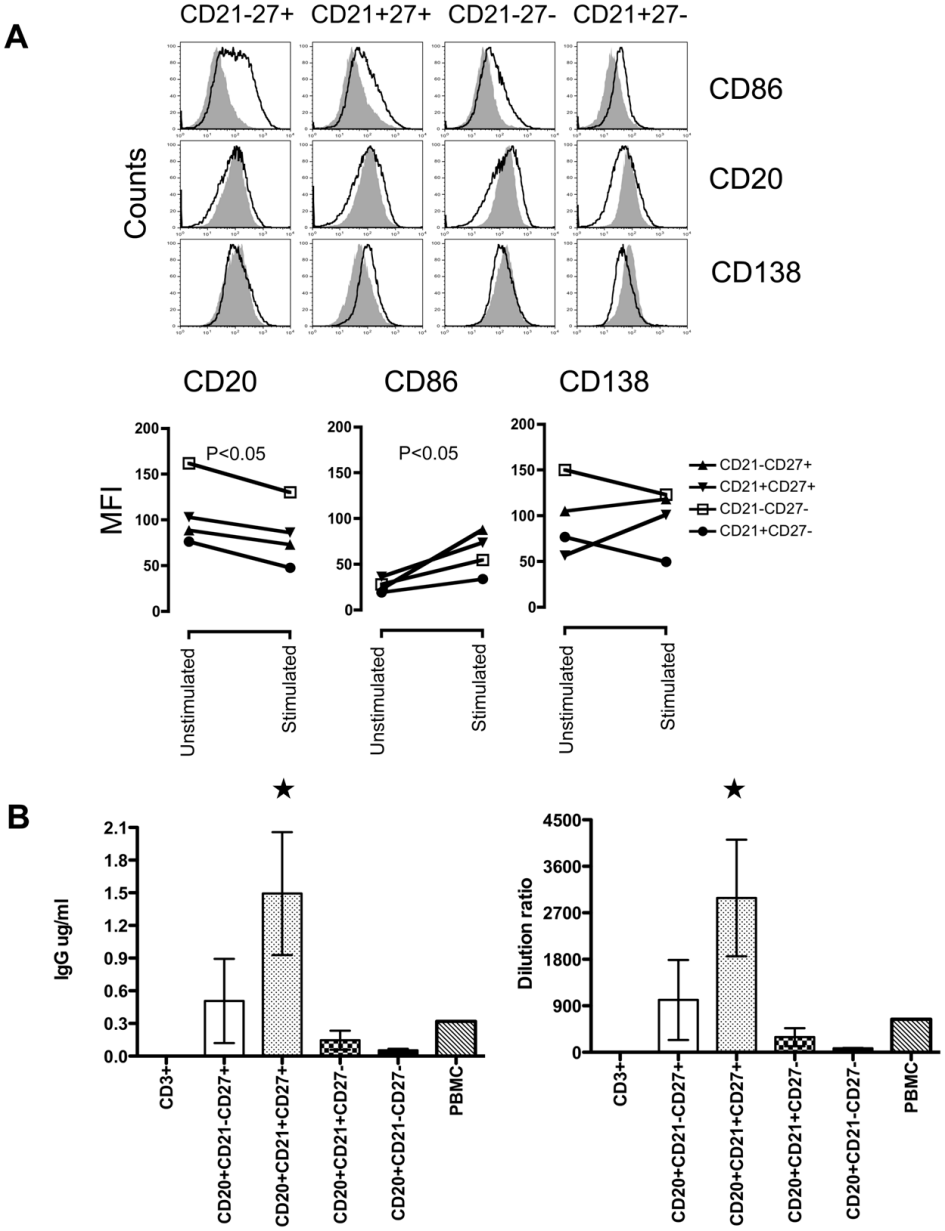
Functional and phenotype properties of B cell subsets. (**A**). All 4 subpopulations (CD21^−^CD27^+^, CD21^+^CD27^+^, CD21^+^CD27^−^, and CD21^−^CD27^−^) of CD3^−^CD20^+^ B cells were sorted from peripheral blood mononuclear cells (PBMC) and stimulated in the presence of pokeweed mitogen, SAC, and CpG-2006 for 6 days. Unstimulated (filled histogram) and stimulated (open histogram) sorted B cell cultures are shown as histogram for expression of anti-CD20, anti-CD138, and anti-CD86. There was significant upregulation of CD86, downregulation of CD20 in all B cell subsets, and upregulation of CD138 expression in DP CD21^+^CD27^+^ B cells. This experiment was repeated independently 3 times and data shown is the mean value of the representative plot. (**B**) Supernatants collected from B cell subsets (CD21^−^CD27^+^, CD21^+^CD27^+^, CD21^+^CD27^−^, and CD21^−^CD27^−^), CD3^+^ T cells and total PBMCs following 6 days stimulation. Sandwich ELISA was performed to determine the level of secretory IgG in culture supernatant. Mean frequency (± standard error) of IgG production is shown for all B cell subsets, T cells and total PBMCs. Increased levels of IgG production were detected in DP CD21^+^CD27^+^ B cells following stimulation compared to other B cell subsets. 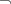 Indicates significant differences between IgG production of DP CD21^+^CD27^+^ B cells and other B cell subsets.

To define which B cell subsets in RMs displayed memory function, we sorted CD20^+^ B cells into four distinct subsets (CD21^−^CD27^+^, CD21^+^CD27^+^, CD21^+^CD27^−^ and CD21^−^CD27^−^). In another sorting experiment, we have sorted CD3^+^ T cell population to compare with all the 4 subsets of B cells as well as with total PBMC. Sorted cells were 95–100% pure for the specified B and T cell populations in all experiments (Figure S1). All the cell subsets were cultured with a polyclonal stimulant cocktail of pokeweed mitogen (PWM), protein A and CpG2006, and observed for changes in cell phenotype by flow cytometry and IgG production in supernatants by ELISA. Six days after polyclonal stimulation a significant decrease in CD20 and increase in CD86 (an activation molecule) mean fluorescent intensity (MFI) was observed in all B cell subsets, yet only DP CD21^+^CD27^+^ B cells showed an increase in CD138 (an useful marker of plasma cell) MFI compared to unstimulated control cultured cells using two tailed student's paired t test analysis (Fig. 2A). There was increased CD138 expression observed in CD21-CD27+ B cells, however, the changes were not statistically significant. There was also a significant increase in IgG production in DP CD21^+^CD27^+^ B cells compared to other cell subsets (P<0.05) (Fig. 2B). The production of IgG in the absence of T cell help suggests that DP CD21^+^CD27^+^ B cells are memory B cells. These results were consistent in 3 independent sorting experiments with PBMC from normal RMs. We also sorted intestinal LPL from 3 independent uninfected RMs and the data were similar to those shown for PBMCs (data not shown).

### Phenotype of DP CD21+CD27+ B cells in peripheral blood and other lymphoid tissues

**Figure 3:**
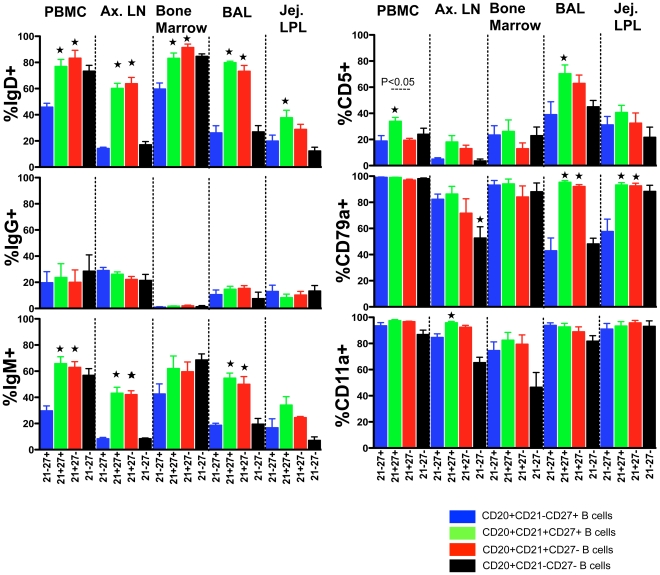
Relative expression of immunoglobulin receptors (IgD, IgG and IgM), other receptors (CD5, CD79a, and CD11a) in different subpopulations of CD20^+^ B cell in both peripheral and lymphoid tissues from normal healthy macaques (mean ± standard error). Cells were gated first on singlets, and then on CD20^+^ B lymphocytes. All CD20^+^ B cells were further analyzed based on their CD21 and CD27 surface molecule expression. Statistically significant differences between DP CD21^+^CD27^+^ and SP CD21^+^ B cell subsets are shown. 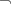 Indicates significant differences between SP CD27^+^ B cells and other B cell subsets for the specified tissue.

The phenotype of DP CD21^+^CD27^+^ memory B cells and other B cell subsets was compared immediately ex vivo in PBMC, ALN, BM, BAL and jejunum LPL tissue samples of 13 normal RMs. Surface immunoglobulin (IgD, IgG and IgM) expression was determined for all four B cell subsets described above. Interestingly, low to negligible amounts of IgG expression was observed in BM B cells. However, IgD and IgM surface expression was significantly higher (p<0.05) in DP memory B cells from PBMC, ALN and BAL tissues compared to SP CD27^+^ B cells (Fig. 3). B-1 cells are a subclass of B cells that express CD5 are produced during fetal development. These cells express greater quantities of IgM, and its receptor shows polyspecificity meaning affinity for immunoglobulins, self-antigens and common bacterial polysaccharides [27]. CD5 expression was higher in DP CD21^+^CD27^+^ B cells in BAL (mean, 70% versus 39%; p<0.05) and jejunum LPL compared to SP CD27^+^ B cells (Fig. 3). Expression of an alternative marker for B cell lineage CD79a, was found to be significantly higher on DP memory B cells in BAL (mean, 95.4% versus 42.9%; p<0.05) and jejunum LPL (mean, 93.2% versus 57.7%; p<0.05) compared to SP CD27^+^ B cells (Fig. 3). However, no significant differences in CD79a were detected in PB cell subsets.

Integrin, alpha L (CD11a) is involved in cellular adhesion and costimulatory signaling. Higher expression of CD11a was prevalent in all B cell subsets from jejunum LPL, PBMC and BAL tissues. However, DP memory B cells in ALN had significantly higher expression of CD11a compared to SP CD27^+^ B cells (P<0.05).

**Figure 4:**
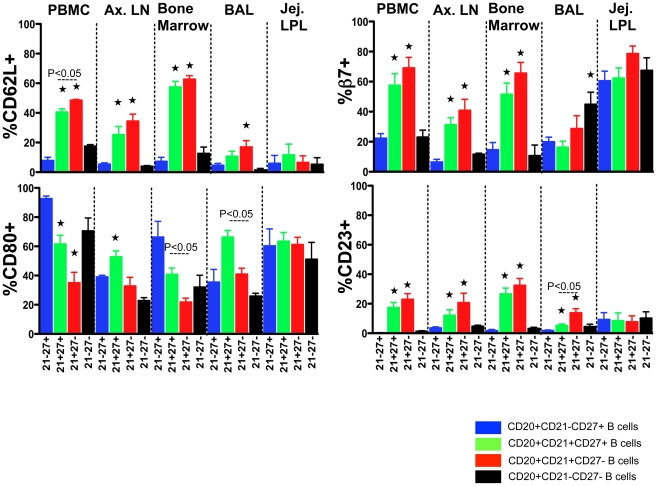
Relative expression of trafficking molecules (CD62L and β7), and activation receptors (CD80 and CD23) in different subpopulations of CD20^+^ B cells in both peripheral and lymphoid tissues from normal healthy macaques (mean ± standard error). Cells were gated first on singlets, and then on CD20^+^ B lymphocytes. All CD20^+^ B cells were further analyzed based on their CD21 and CD27 surface molecule expression. Statistically significant differences between DP CD21^+^CD27^+^ and SP CD21^+^ B cell subsets are shown. 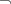 Indicates significant differences between SP CD27^+^ B cells and other B cell subsets for the specified tissue.

Higher expression of CD62L [28], a peripheral lymph node homing molecule, was detected on DP memory B cells from PBMC, ALN and BM tissues (P<0.05) compared to SP CD27^+^ B cells. Moreover, CD62L expression was much lower in both mucosal tissues (BAL and jejunum LPL) on all four B-cell subsets, consistent with its role as a peripheral addressin (Fig. 4).

CD80 (B7.1) is a costimulatory molecule involved in T cell activation and survival. CD80 was predominantly expressed on SP CD27^+^ B cells from PBMC and BM. Increased CD80 expression was also detected on DP CD21^+^CD27^+^ B cells from tissues like ALN and BAL (Fig. 4).

The integrin α4β7 mediates lymphocyte migration to the intestine through interaction with the mucosal addressin cell adhesion molecule-1 (MAdCAM-1), which is predominantly expressed on venules in the gut-associated lymphoid tissue and intestinal LPL [29]. To measure intestinal homing cells, we examined β7 expression on B cell subsets [30]. In jejunum LPL, all B cell subsets had high levels of β7 expression (means, 60.6%–78.6%). However, DP memory B cells had significantly higher expression of β7 compared to SP CD27^+^ B cells from PBMC, ALN and BM (Fig. 4).

CD23, the low-affinity IgE receptor is widely distributed on cells and mediates IgE-related immune responses by enhancing IgE antigen complex presentation, regulating IgE synthesis and influencing cell differentiation and growth of both B and T cells. Higher expression of CD23 was detected on DP CD21^+^CD27^+^ and SP CD21^+^ B cells in PBMC (mean value range, 17.5%–23.0%), ALN (mean value range, 12.2%–20.8%), and BM (mean value range, 26.7%–32.6%) compared to SP CD27^+^ B cells. Expression of CD23 was lower in all B-cell subsets isolated from BAL and jejunum LPL tissues (Fig. 4).

### Increased rate of lymphocyte turnover in DP CD21^+^CD27^+^ B cells

**Figure 5:**
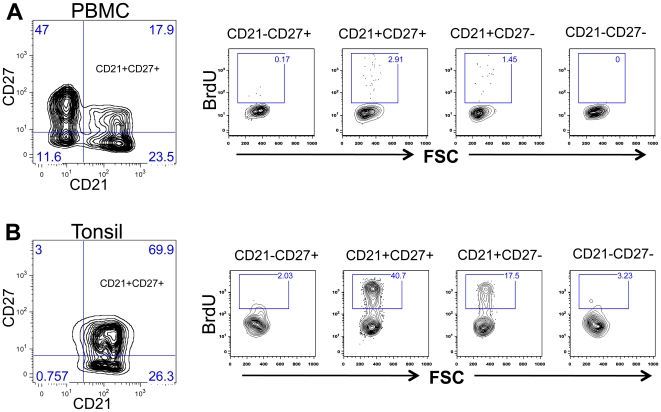
Representative flow cytograms show increased cell turnover (BrdU^+^) in DP CD21^+^CD27^+^ B cells in peripheral blood (A) and tonsillar (B) lymphocytes from a normal healthy uninfected rhesus macaque (GN74). Singlets were gated first to eliminate doublets and finally gating was performed on CD20^+^ B lymphocytes. All CD20^+^ B cells were further analyzed based on their CD21 and CD27 surface molecule expression. The percentage of BrdU^+^ cells is indicated in the top box of each panel. Note that DP CD21^+^CD27^+^ B cells had higher BrdU^+^ proliferating cells than other B cell subsets.

**Figure 6:**
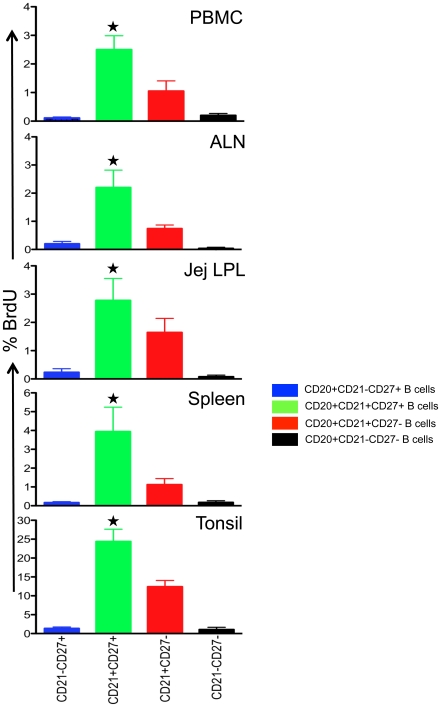
Bar graphs showing the mean BrdU^+^ proliferative responses (± standard error) in different CD20^+^ B cell subsets from different tissues of normal macaques. Singlets were gated first to eliminate doublets and finally gating was performed on CD20^+^ B lymphocytes. All CD20^+^ B cells were further analyzed based on their CD21 and CD27 surface molecule expression. BrdU was injected intraperitoneally and tissues were collected 24 hrs after inoculation. Increased proliferation of DP CD21^+^CD27^+^ B cell subsets compared to other B cells subsets were observed in all healthy normal rhesus macaques. *Indicates significant differences between BrdU expression of DP CD21^+^CD27^+^ B cells and other B cell subsets.

Because more DP CD21^+^CD27^+^ B cells had the capacity to produce antibodies following polyclonal stimulation (and without T cell help), we hypothesized DP CD21^+^CD27^+^ B cells would have higher rates of proliferation than their SP counterparts. To test this, 10 normal RMs were inoculated with bromodeoxyuridine (BrdU) 24 hrs prior to sampling to detect cells in S-phase (DNA synthesis) of division in tissues like PBMC, ALN, Jejunum LPL, Spleen and Tonsil. Overall, DP CD21^+^CD27^+^ B cells demonstrated the highest level of BrdU incorporation in all tissues (mean, 2.3%–24.1%; p<0.05) followed by SP CD21^+^CD27^−^ B cells (mean, 0.76%–12.3%), and SP CD21^−^CD27^+^ B cells (mean, 0.1%–1.34%; Figs 5 & 6). Double negative CD21^−^CD27^−^ B cells had a lowest proliferating B cells population compared to all the B cell subsets. Moreover, tonsillar B cells (particularly DP CD21^+^CD27^+^ and SP CD21^+^CD27^−^ B cells) had higher percentages of BrdU incorporation (mean, 12.3–24.1%; p<0.05) compared with all other tissues examined (mean, 0.76–3.94%). Combined, these data indicate that in all tissues, DP CD21^+^CD27^+^ B cells have much higher rates of proliferation than SP cells.

## Discussion

Direct interactions between HIV and B cells were reported earlier, where HIV binds to B cells in vivo through complement receptor CD21 [31], [32], [33], although there is little evidence that HIV can productively replicate in B cells in vivo. Several B-cell functional abnormalities have also been associated with HIV/SIV infection, including hypergammaglobulinemia [20], [21], [22], increased basal activation and reduced sensitivity to mitogen stimulation [26], [34], [35], and depletion of the CD27^+^ memory B-cell subset [20], [21], [22], [23], [35]. The mechanism by which memory B cells are lost during acute phase of SIV/HIV infection remains unclear. The loss of memory B cells was shown to be positively correlated with the loss of CD4+ T cells in the periphery [23]. However other researchers have conflicting evidence to support the lack of correlation between memory B calls and CD4+ T cells count in chronic HIV infection [24]. The immune activation following SIV infection may also predispose these memory cells to activation-induced apoptosis. The ability of memory B cells to generate a robust and efficient antibody response to secondary challenge by an invading pathogen is a hallmark of immunological memory. Indeed, several in vivo and in vitro studies have demonstrated that CD27^+^ memory B cells proliferate and differentiate into immunoglobulin secreting effector cells more rapidly than do naïve B cells [5], [6], [36], [37]. To define which subset of CD27^+^ B cells act as memory B cells, we examined all CD20^+^ B cells based on CD27 and CD21 surface expression and their functional characteristics (e.g. IgG production). Bernasconi and his group previously showed that long term serological memory could be generated by antigen independent polyclonal activation and differentiation of memory B cells [3]. In this study we utilized T cell-independent polyclonal stimulation to define memory B cell function. Our results demonstrate that DP CD21^+^CD27^+^CD20^+^ B cells are memory B cells that can be activated and induced to proliferate and differentiate into antibody secreting cells via polyclonal activation without T cell help. Similarly, SP CD21^−^CD27^+^, SP CD21^+^CD27^−^, DN CD21^−^CD27^−^ B cells are believed to be naïve or in a transitional memory stage, as they are less efficient in antibody production following polyclonal stimulation without T helper cells. Double negative CD21^−^CD27^−^ B cells isolated from human peripheral blood have also been reported recently as tissue like memory B cells, with low in vitro proliferative responses compared to other B cells [38]. We have also shown increased DP CD21^+^CD27^+^ memory B cell populations exist in all lymphoid and mucosal tissues (secondary lymphoid tissues) compared to BM and PBMC, which is also consistent with their role as memory B cells.

DP CD21^+^CD27^+^ B cells demonstrated a marked increase in surface immunoglobulin (IgD and IgM) expression, mucosal homing and cell adhesion (β7), peripheral homing (CD62L) and activation (CD23) compared to SP CD27^+^ B cells, which indicate these two B cell subsets differ phenotypically. B-1 (CD5) cells are believed to be the primary source of IgM production [39]. DP memory B cells also had increased CD5 expression in parallel to increased IgM expression. Increased CD23 expression on DP CD21^+^CD27^+^ memory B cells in tissues also supports the earlier observation where CD23 expression has been correlated with enhanced antibody responses [40] and its increased expression on activated B cells [8]. It has also been shown that memory B cells express the gut homing receptor α4β7 [41], which also was true for the DP CD21^+^CD27^+^ B cells isolated from PBMC, ALN, BM. Increased CD62L expression in DP CD21^+^CD27^+^ and SP CD21^+^ B cells also suggest that these cells may be highly motile travelling around the system and if necessary modulate the immune response. Both DP CD21^+^CD27^+^ and SP CD21^+^CD27^−^ cells had similar phenotypic expression of immunoglobulin receptors (IgD, IgM and IgG), CD11a integrin, and CD79a molecules. There was increased CD23, β7 and CD62L and decreased CD5 and CD80 surface expression in SP CD21^+^CD27^−^ cells compared to DP CD21^+^CD27^+^ B cells, however differences were not statistically significant in most of the tissues. This is suggestive of similar activation, cell adhesion and homing properties between DP CD21^+^CD27^+^ and SP CD21^+^CD27^−^ B cells. However, their were functional differences in antibody production and cell proliferation between these two B cell subpopulation suggesting DP CD21^+^CD27^+^ B cells are memory cells and SP CD21^+^CD27^−^ cells are in their naïve or transitional memory stage.

The proliferative capacity of naïve and memory B cells was found to be distinct when given an identical stimulus [36]. By modeling the rate of BrdU uptake in vivo in normal adult RMs, we were able to study the differences in lymphocyte turnover rates among B-cell subsets. Despite higher percentages of SP CD21^−^CD27^+^ B cells in PB, the DP CD21^+^CD27^+^ memory B cells had the highest turnover rate, which also suggests that they are more activated, differentiated, functional, and have the characteristics of memory cells. High rates of DP CD21^+^CD27^+^ B cell turnover may help to maintain the pool of functional B cell populations in normal RMs.

To our knowledge, this is the first report on cycling properties of different B cell subsets, and for identifying a potential role for DP CD21^+^CD27^+^ B cells as memory B cells in primates. The DP B cells were capable of producing antibody in absence of T cell help, and had increased cell turnover compared to other B cell subpopulations examined. In summary, the data presented in this study clearly demonstrate that the DP CD21^+^CD27^+^ B cells are memory cells and are highly proliferative than other B cell subsets. Tonsil has the highest proliferating memory B cell population than any other tissues examined. It is important to monitor the proliferating and functional capabilities of these specific B cell subsets during SIV infection to fully understand the extent of the damage to the memory B cell and correlate these effects with antigen specific humoral immune responses.

## Materials and Methods

### Ethics statement

Approval for all veterinary procedures in this study had been obtained from the Institutional Animal Care and Use Committee (Protocols #322), Animal Welfare Assurance A-4499-01. All the animals in this project were housed in Tulane National Primate Research Center (TNPRC) and under the full care of TNPRC veterinarians in accordance with the standards incorporated in the Guide to the Care and Use of Laboratory Animal (NIH) 78-23 (Revised, 1996). All veterinary procedures were performed only with sedated animals.

### Animals, BrdU, and tissue sampling

Thirteen female Indian RMs (*Macaca mulatta*) between 3–16 years of age, which were initially negative for HIV-2, SIV, type D retrovirus and STLV-1 infection were used in this study. Only 10 macaques were intraperitoneally inoculated with nucleotide analog bromodeoxyuridine (BrdU; 60 mg/kg in sterile saline, Sigma-Aldrich, St Louis, MO) 24 hrs prior to euthanasia and tissue collection [42]. EDTA anti-coagulated blood, ALN, tonsil, spleen, BM, BAL and intestines (jejunum) were collected at necropsy for functional and/or phenotyping experiments.

### Lymphocyte isolation from tissues

Lymphocytes from the PB, intestine, ALN, and spleen were isolated as previously described [42], [43], [44], [45], [46]. In brief, intestinal epithelial lymphocytes were separated from intestinal pieces by incubating 1-cm^2^ pieces of tissue in EDTA with rapid shaking at 37°C. Mucus and large debris were removed from the supernatant by filtering through loosely packed glass wool. After epithelial removal, LPL were collected by mincing the remaining tissue into 1–2 mm pieces, followed by digestion in complete RPMI 1640 medium (RPMI-5) containing 60 units/ml of Type II collagenase (Sigma-Aldrich) again with rapid shaking at 37°C. For enrichment of lymphocytes, supernatants of LPLs were centrifuged over discontinuous Percoll (Sigma-Aldrich) density gradients followed by washing with phosphate-buffered saline (PBS). For lymphocyte isolation from ALN, tonsil and spleen, tissues were simply minced and gently pressed through 70-um nylon cell strainers. All cells were washed twice and resuspended in complete RPMI-5 medium containing 5% fetal calf serum (FCS) before staining. Bronchoalveolar lavage samples were collected in normal saline solution. Immediate after collection FCS was added to the collection tubes at the concentration of 10% and was mixed thoroughly. The cells were then resuspended in complete RPMI-10 medium containing 10% FCS after washing. Bone marrow cells were filtered, lysed with ACK solution (Lonza Biowhittaker) and counted for further immunofluorescent staining. All lymphocytes were >90% viable by trypan blue dye exclusion method.

### Single cell sorting

Fresh PBMCs were isolated from normal healthy RM. PBMCs were stained for flow cytometric analysis using combinations of the following fluorochrome-conjugated monoclonal antibodies: CD3-Pacific Blue (SP34.2), CD20-PE (L27), CD21 PE-Cy5 (B-Ly4), and CD27-FITC (M-T271) obtained from BD Biosciences. Unfixed live cells were sorted four-ways using a FACS-Aria cell sorter instrument, and at least 1.5×10^6^ cells were sorted for each subset of cells. Sorted cells were further washed and resuspended in RPMI-10 media and adjusted to 10^7^ cells/ml. 0.5×10^6^ cells per well were cultured in 24 well plates in the presence of PWM (kindly provided by Dr. Shane Crotty, La Jolla Institute for Allergy and Immunology, CA), Protein A (SAC, Sigma-Aldrich), β-mercaptoethanol (Sigma-Aldrich) and CpG-2006 oligonucleotides (Operon technologies) for 6 days at 37°C in presence of 5% CO_2_ as described for human memory B cell stimulation [47]. Following stimulation, ELISA was performed to quantify IgG production (see below) with the supernatant collected from each well. Cells from each well were resuspended in staining media and further stained for CD86 (activation), CD138 (plasma cell) and CD20 phenotyping markers. Unstimulated cells were kept as negative controls for each experiment and a total of 3 independent experiments were performed with sorted cells.

### Quantitation of immunoglobulins

Total IgG levels in cell culture supernatants were measured by sandwich ELISA as described earlier [48]. In brief, plates were coated with goat anti-monkey IgG Fc at 6 ug/ml in PBS (Accurate Chemicals, NY) and incubated overnight at 4°C. The IgG standard was obtained from National Institutes of Health (NIH) Nonhuman Primate Reagent Resource (NPRR). Cell culture supernatants were diluted as necessary and tested in duplicate. The IgG was detected with peroxidase-conjugated goat anti-monkey IgG secondary antibody (Accurate Chemicals). Plates were then developed with o-phenylenediamine dihydrochloride (OPD) (Sigma-Aldrich) for 5 min and stopped with 2N H_2_SO_4_, and the absorbance was measured on a spectrophotometer at 490 nm with a reference filter of 650 nm. The specificity of the rhesus monkey IgG detection antibody has been previously demonstrated [49]. The IgG concentrations in cell culture supernatants are presented as micrograms per milliliter.

### Immunofluorescent staining and flow cytometric analysis

For flow cytometry staining, cells were adjusted to 10^7^ cells/ml and 100 ul aliquots or 100 ul of whole blood samples were incubated with appropriately diluted concentrations of antibodies for 30 min at 4°C. Whole blood, and spleen samples were then lysed and washed using a whole blood lysis protocol as previously described [43], [50], [51]. Stained cells were then washed once with PBS and fixed with 1X BD stabilizing fixative buffer (BD Biosciences). For BrdU staining, the cells were first surface stained, then fixed and permeabilized using Cytofix/Cytoperm (BD Biosciences), washed in Perm Buffer (BD Biosciences), incubated 30 min at room temperature with FITC-conjugated anti-BrdU in Perm Buffer, washed, and resuspended in 1X BD stabilizing fixative buffer (BD Biosciences) [42]. Cells were kept protected from light at 4°C and acquisition was performed within 24 hrs of staining. Lymphocytes from ALN, intestinal LPL, BM, and tonsil were stained and processed similar to blood tissues with the omission of the whole blood lysing technique [51]. Polychromatic (9–11 parameter) flow cytometric acquisition was performed on a Becton Dickinson FACS-Aria or LSR II instrument with three lasers (488 nm blue laser, 633 nm red laser and 407 violet laser) using FITC, PE, PE-Texas red, PE-Cy5, PerCP-Cy5.5, APC, Alexa 700, APC-Cy7, Pacific Blue, and Qdot655 as the available fluorochrome parameters. Single-stained controls for each fluorochrome were used for compensation settings. Monoclonal antibodies CD3 (SP32-2), CD11a (HI111), CD20 (L27), CD21 (B-ly4), CD23 (M-L233), CD27 (M-T271), CD62L (SK11), CD79a (HM47), CD80 (L307.4), CD138 (MI15), IgG (G18-145), IgM (G20-127), β7 (FIB504), and BrdU FITC (3D4) were obtained from BD Biosciences. CD5 (CD5-5D7), CD8 (MHCD0817) and CD4 Qdot655 (T4/19Thy5D7) were obtained from Invitrogen and the NIH NPRR courtesy of Dr. K. Reimann (Harvard University, Cambridge, MA) respectively. IgD (purified polyclonal) and CD27 (0323) were obtained from Southern Biotech and eBioscience respectively.

At least 30,000 events were collected from each sample by gating on lymphocytes and data were analyzed using FlowJo software (TreeStar Inc.) version 8.8.6. All monoclonal antibodies were first titrated and tested for their crossreactivity in RMs, for both T and B cells.

### Statistics

Results of experimental groups were compared using either a two-tailed Student's paired t-test or nonparametric Mann-Whitney t test using Prism software (GraphPad software, SanDiego, CA). P values <0.05 were considered significant.

## Supporting Information

Figure S1 **Representative dot plots showing single cell sorting of peripheral CD3^+^ T and CD20^+^ B cells from a normal rhesus macaque using 4-way sort FACSAria instrument.** Post sort confirmation shows that all the 4 different T and B cell subpopulation have 95-100% purity. Singlets were gated first to eliminate doublets and finally gating was performed on CD3^+^ T or CD20^+^ B lymphocytes. CD20^+^ B cells were further gated to define 4 different subpopulations (CD21^−^CD27^+^, CD21^+^CD27^+^, CD21^−^CD27^−^ and CD21^+^CD27^−^) based on CD21 and CD27 phenotype markers.
(TIF)
