# Supplementary figures and images for: Double-Positive CD21+CD27+ B Cells Are Highly Proliferating Memory Cells and Their Distribution Differs in Mucosal and Peripheral Tissues
Source: PLoS One. 2011 Jan 27;6(1):e16524. doi: 10.1371/journal.pone.0016524 (PMC3029363; doi:10.1371/journal.pone.0016524)

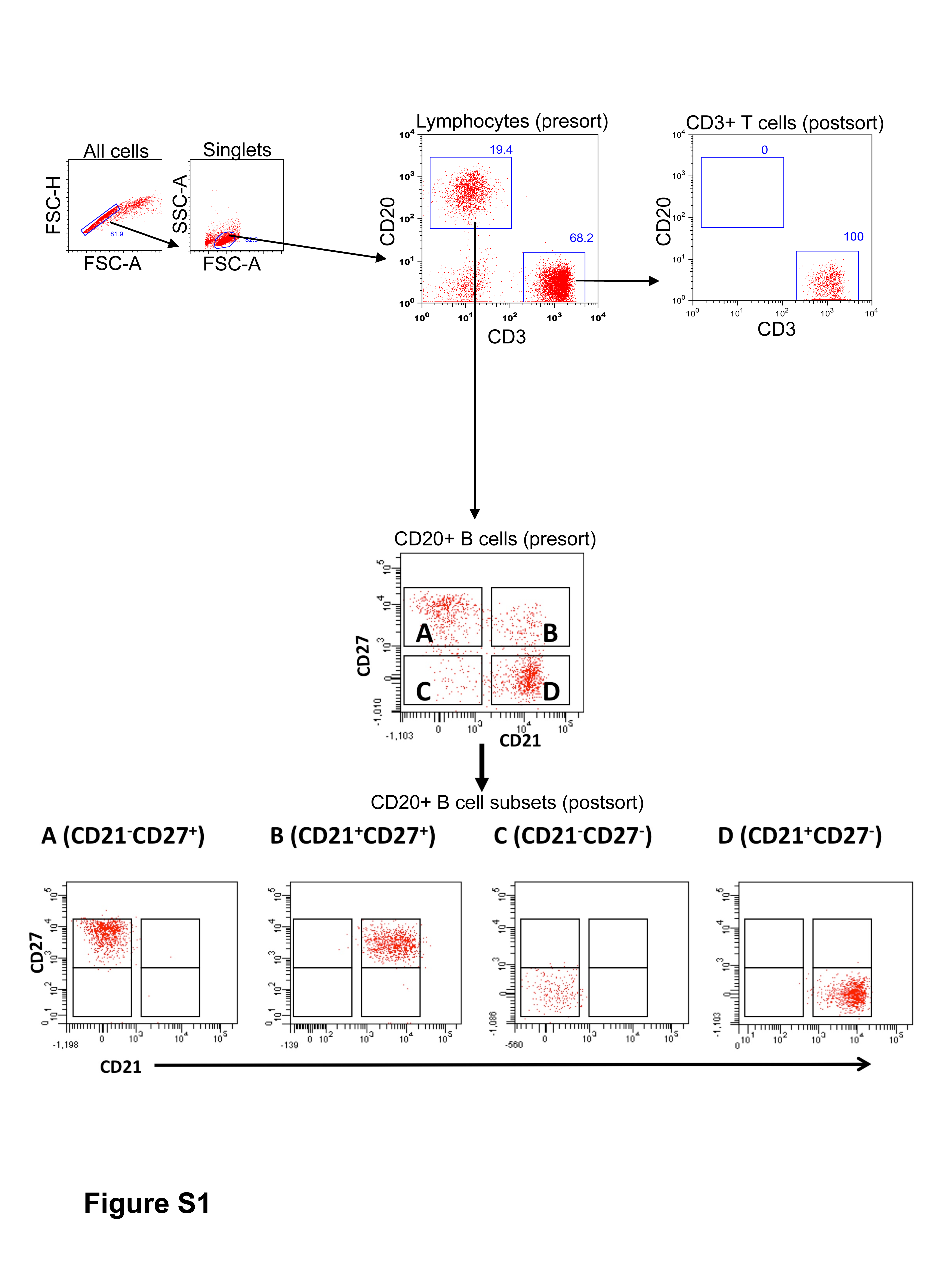

Supplement: Figure S1 — Representative dot plots showing single cell sorting of peripheral CD3+ T and CD20+ B cells from a normal rhesus macaque using 4-way sort FACSAria instrument. Post sort confirmation shows that all the 4 different T and B cell subpopulation have 95-100% purity. Singlets were gated first to eliminate doublets and finally gating was performed on CD3+ T or CD20+ B lymphocytes. CD20+ B cells were further gated to define 4 different subpopulations (CD21−CD27+, CD21+CD27+, CD21−CD27− and CD21+CD27−) based on CD21 and CD27 phenotype markers. (TIF) [file pone.0016524.s001.tif]
